# Mechanisms and disease implications of sirtuin-mediated autophagic regulation

**DOI:** 10.1038/s12276-019-0302-7

**Published:** 2019-09-06

**Authors:** In Hye Lee

**Affiliations:** Department of Life Science, Ewha Womans University, Seoul, South Korea

**Keywords:** Cancer metabolism, Cell signalling

## Abstract

Accumulating evidence has indicated that sirtuins are key components of diverse physiological processes, including metabolism and aging. Sirtuins confer protection from a wide array of metabolic and age-related diseases, such as cancer, cardiovascular and neurodegenerative diseases. Recent studies have also suggested that sirtuins regulate autophagy, a protective cellular process for homeostatic maintenance in response to environmental stresses. Here, we describe various biological and pathophysiological processes regulated by sirtuin-mediated autophagy, focusing on cancer, heart, and liver diseases, as well as stem cell biology. This review also emphasizes key molecular mechanisms by which sirtuins regulate autophagy. Finally, we discuss novel insights into how new therapeutics targeting sirtuin and autophagy may potentially lead to effective strategies to combat aging and aging-related diseases.

## Age-related diseases: Protective protein potential

Harnessing the protective powers of a family of proteins could help tackle age-related diseases. In a review of recent research, In Hye Lee at Ewha Womans University in Seoul, South Korea, explores links between the sirtuin protein family and autophagy, a natural process involving the degradation and recycling of cellular components. In mammals, the sirtuin family comprises seven proteins that are found in specific locations across the body, and play key roles in many biological processes from metabolism to the regulation of autophagy. As we age, autophagic balance becomes disrupted, leaving us vulnerable to diseases such as cancer and neurodegenerative disorders. Indeed, cancerous tumors can exploit autophagic processes for their own benefit. Lee’s review suggests that therapies targeting sirtuins could restore and maintain autophagic balance, potentially helping to control aging and age-related disorders.

## Introduction

Sirtuins act predominantly as NAD^+^-dependent deacetylases for a wide range of target proteins, which are crucial for various biological processes. Sir2 (silent information regulator 2) was the first sirtuin to be discovered, and numerous studies have demonstrated that Sir2 is critical for transcriptional silencing in budding yeast^1^. The increased expression of yeast Sir2 and its invertebrate orthologs delay aging, increase lifespan and may mediate the effects of dietary restriction^2^.

Mammals have seven sirtuin proteins, denoted as SIRT1 through SIRT7, each of which exhibits distinct subcellular localization and rather specific functions. These seven mammalian sirtuins regulate diverse physiological processes ranging from metabolism to epigenetic modification in many tissues^3–6^. SIRT1 and SIRT2 are localized to both the nucleus and cytoplasm^7^. SIRT1, SIRT6, and SIRT7, which are mainly localized to the nucleus, deacetylate intracellular signaling proteins, including histones, and regulate gene expression^5,8^. SIRT2 can act as a nuclear protein and regulate the cell cycle, although SIRT2 is mainly localized to the cytoplasm^5,9,10^. SIRT3, SIRT4, and SIRT5 are exclusively localized to the mitochondria and regulate energy metabolism in response to mitochondrial stress^4,7,11^. For instance, SIRT3 deacetylates various proteins to regulate amino acid metabolism, fatty acid oxidation, the tricarboxylic acid (TCA) cycle, electron transport chain (ETC) activity, mtDNA replication, transcription, and translation. Many target proteins of SIRT4 overlap with proteins that are regulated by SIRT3. SIRT5 specifically interacts with several enzymes crucial for fuel utilization and energy production^4^. Overall, mitochondrial sirtuins regulate mitochondrial protein networks, orchestrate mitochondrial function, and allow cells to adapt to metabolic stresses. In addition, emerging evidence indicates that sirtuins regulate yet another important cellular process, autophagy.

Autophagy is a process by which damaged proteins and organelles, including damaged mitochondria, endoplasmic reticulum (ER), and peroxisomes, are targeted for lysosomal-mediated degradation. This autophagy-mediated degradation process is useful for removing damaged cellular components and for providing metabolic intermediates necessary for protein synthesis and metabolism. When cells are stressed, autophagosome formation from the phagophore is induced. This de novo formation of autophagosomes likely derives from existing organelles such as the ER or mitochondria^12,13^. Autophagosomes then elongate, and the double membranous autophagosome encapsulates its damaged cargo, eventually fusing with the lysosome. This cellular process is, as mentioned above, induced by a number of stresses, including nutrient withdrawal, a classic inducer of autophagy. Nonetheless, autophagy is also essential for basal homeostatic maintenance, as shown in animal models in which autophagy is disrupted in a tissue-specific fashion, leading rapidly to the massive accumulation of damaged proteins and organelles within autophagy-deficient tissues^14,15^.

In this review, we focus on recent progress regarding sirtuin-mediated autophagy and its physiological importance in human biology.

## The regulation of autophagy by various sirtuins

### The regulation of autophagy by SIRT1

**Fig. 1:**
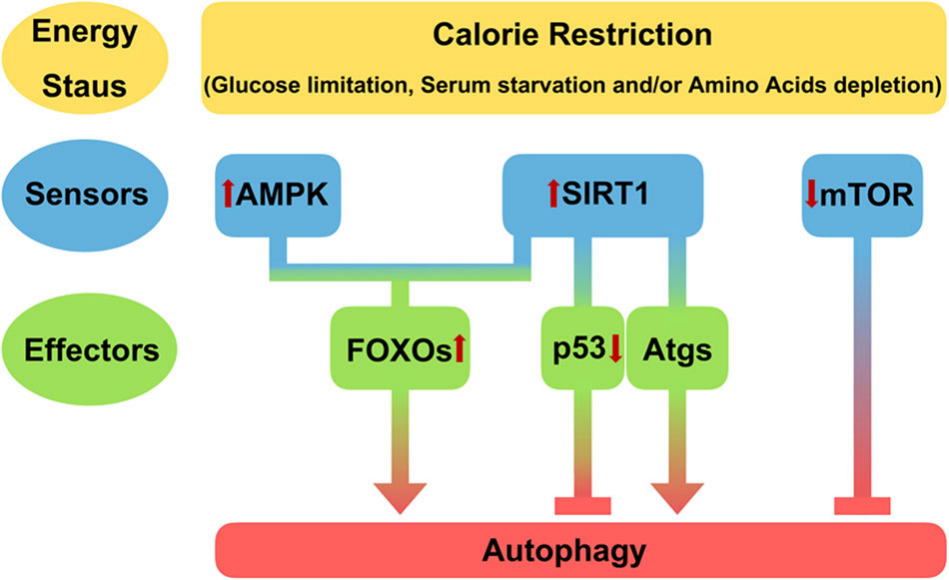
The regulation of autophagy by SIRT1 under calorie restriction. The level of nutrients (e.g., glucose, lipids, and amino acids) is detected by energy sensors, namely, AMPK, SIRT1, or mTOR^17,97^^,104^. Calorie restriction activates AMPK or SIRT1 but inhibits mTOR activity. AMPK and SIRT1 upregulate the expression of Atgs through the activation of forkhead box transcription factors (FOXOs)^97^^,105^. In addition, SIRT1 also forms a molecular complex with Atg proteins, including Atg7 and Atg8 (LC3). SIRT1 deacetylates the core autophagy machinery in nutrient restricted conditions and thereby increases autophagy. Calorie restriction also potentiates the expression of SIRT1 and increases its deacetylase activity^17,18^. SIRT1 deacetylates p53^18^, which downregulates p53 and increases autophagy^19,20^. However, another well-known negative regulator of autophagy, mTOR, is downregulated under low nutrient conditions

Both autophagy and sirtuins appear to protect cells from environmental stresses, including nutrient stress^14,16^ (Fig. 1). Essentially, cells induce autophagy in response to nutrient withdrawal. Starvation increases the levels of SIRT1 in mammalian cells, as well as in certain mouse tissues. This SIRT1 transcriptional induction requires the forkhead transcription factor FOXO3a, which forms a complex with p53 in a nutrient-sensitive manner^17^. In addition, calorie restriction protects mammalian cells from apoptosis^18^. Calorie restriction increases the level of SIRT1^18^, which can deacetylate targets, including the DNA repair factor Ku70 and p53^18^. The deacetylation of p53 by SIRT1 suppresses the activity of p53 in essential physiological roles such as apoptosis and induces autophagy^19,20^ (Fig. 1). Cytoplasmic p53 inhibits autophagy in a transcription-independent manner^19,21,22^(Fig. 1), but nutrient depletion, a physiological inducer of autophagy, decreases p53 expression by activating the ubiquitin E3 ligase Mdm2 and activates autophagy^23^. SIRT1 overexpression also decreases p53 expression and protects cells from apoptosis^20^. However, p53 has dual roles in autophagy depending on its subcellular localization. For example, nuclear p53 induces autophagy^21,22,24^, and the molecular mechanisms by which p53 regulates autophagy need to be further clarified.

Under cellular stress, including nutrient depletion, SIRT1 activity is increased, autophagy proteins are deacetylated, and subsequently autophagy is induced^25^ (Fig. 1). While the increase in sirtuin activity is likely multifactorial, the relationship between caloric restriction and a rise in NAD^+^ levels is clearly one attractive hypothesis that connects nutrient deprivation, sirtuin activation and autophagy induction^18,25–27^. Several autophagy proteins (Atgs), including Atg7 and Atg8, directly interact and are deacetylated by SIRT1^25^. Furthermore, *Sirt1*-deficient mice display increased basal acetylation of essential autophagic proteins and decreased autophagy function. This defective autophagy is particularly striking given the apparent accumulation of damaged mitochondria in *Sirt1-*deficient heart tissues^25^.

Another hallmark of mammalian autophagy is an increase in the number of LC3 (autophagy-related protein 8, Atg8) puncta, as measured by using a fusion protein consisting of green fluorescent protein (GFP) and LC3 (GFP-LC3). This fusion protein is often used as a marker of autophagosome formation. Starvation increases the translocation of GFP-LC3 from the nucleus to the cytoplasm in wild-type mouse embryonic fibroblasts (MEFs), indicating the stimulation of autophagy under nutrient-depleted conditions^25^. However, in *Sirt1*-deficient cells, GFP-LC3 puncta formation is highly reduced. The expression of wild-type *SIRT1* in *Sirt1*-deficient MEFs restores autophagy in these cells, while a deacetylase-inactive mutant of *SIRT1* is unable to restore autophagic activity^25^. Thus, the deacetylase activity of SIRT1 is required for the stimulation of autophagy in nutrient-depleted cells. In addition to these loss-of-function experiments, many lines of evidence suggest that the increased expression of SIRT1 is sufficient to induce autophagy by promoting the conversion of LC3-I to LC3-II through proteolytic cleavage and lipidation^25^.

How does SIRT1 regulate autophagy? SIRT1 forms molecular complexes with essential components of the autophagy machinery, including Atg5, Atg7 and LC3. SIRT1 deacetylates these proteins in cultured cells and neonatal tissues^25^. As mentioned above, the genetic inhibition of *SIRT1* markedly increases the acetylation of endogenous Atg7 in various tissues, including the heart, brain and liver. The level of Atg7 acetylation is also elevated by treatment with nicotinamide, a pharmacological sirtuin inhibitor. Moreover, purified wild-type SIRT1, but not a catalytically inactive point mutant, can deacetylate Atg7 in an NAD-dependent manner in vitro^25^.

These initial studies have established that starvation activates SIRT1, which in turn deacetylates the autophagic components to augment autophagy. Subsequent studies have delineated additional aspects of this process. In particular, the deacetylation of nuclear LC3 drives autophagy initiation under starvation^28^. Upon nutrient depletion, SIRT1 deacetylates nuclear LC3 at lysine residues K49 and K51, which leads to the interaction between LC3 and the nuclear protein DOR (diabetes- and obesity-regulated nuclear factor), which is required for the translocation of both LC3 and DOR to the cytoplasm. LC3 then binds to Atg7 and other autophagy factors and subsequently undergoes phosphatidylethanolamine conjugation to form a preautophagic membrane structure^28^.

SIRT1-mediated autophagy is inhibited by REGγ, a proteasome activator, which regulates the stability of SIRT1 under starvation conditions^29^. Under normal nutrient conditions, REGγ mediates the ubiquitin-independent degradation of SIRT1, which inhibits autophagy by preventing SIRT1 from binding and deacetylating the autophagy complex components. Thus, REGγ deficiency causes dramatic autophagy induction. Similarly, REGγ dissociates from SIRT1 in response to energy depletion (GD, glucose deprivation), and this contributes to the induction of autophagy because SIRT1 can then interact with the autophagic machinery. GD reduces the interaction between REGγ and SIRT1, at least in part through the phosphorylation of SIRT1 at a specific threonine residue (T530). Evidence suggests that this phosphorylation is mediated by AMP-activated protein kinase (AMPK)^29^. Treatment with compound C, an AMPK inhibitor, reduces GD-induced SIRT1 phosphorylation and inhibits the dissociation of SIRT1 from REGγ. Conversely, treatment with AICAR, an AMPK activator, decreases the interaction between REGγ and SIRT1 and in turn activates autophagy^29^.

### The regulation of autophagy by SIRT2

Recent reports have suggested that SIRT2 inhibits autophagy under basal conditions^30,31^. Specifically, SIRT2 binds and deacetylates FOXO1 under basal conditions. However, FOXO1 is highly acetylated when autophagy is induced in response to serum starvation or oxidative stress. This is because cytosolic FOXO1-SIRT2 complexes dissociate in response to oxidative stress or serum starvation, leading to an increase in the acetylation of FOXO1. Under *SIRT2* deficiency, acetylated FOXO1 interacts with Atg7, which appears to be required for the induction of autophagy^30^. These observations stand in contrast to the results described for SIRT1, for which the levels of Atg protein acetylation under *SIRT1* deficiency^25^ are negatively correlated with autophagic flux^25,32^.

In addition, other observations have suggested that SIRT2 regulates mitochondrial protein deacetylation, mitophagy, and mitochondrial function^33–35^. The depletion of *Sirt2* results in an increase in the acetylation of mitochondrial proteins, morphological changes to mitochondria, increases in oxidative stress and decreases in ATP production. Dysfunctional mitochondria need to be cleared by mitophagy, a selective type of autophagy that specifically removes damaged mitochondria. The loss of *Sirt2* appears to cause defects in mitophagy, as increased levels of p62, PINK1/Parkin, and ubiquitin, are detected in the brains of mice that lack *Sirt2*. In addition, autophagy is impaired in *Sirt2*-deficient MEFs in response to external stimuli. Moreover, the chemical uncoupler carbonyl cyanide-4-(trifluoromethoxy) phenylhydrazone (FCCP) induces mitophagy in wild-type MEFs but not in *Sirt2*-deficient MEFs^33^.

### The regulation of autophagy by SIRT3

SIRT3 is a mitochondrial sirtuin and is responsible for the deacetylation of various mitochondrial proteins, thereby regulating ATP generation, beta oxidation, and urea cycle activity^36,37^. SIRT3 also regulates hepatic autophagy in a manner that is distinct from that of SIRT1^36^. In particular, SIRT3 acts as a negative regulator of autophagy with respect to lipid overload. Indeed, the levels of SIRT3 correlate with the susceptibility of hepatocytes to lipotoxicity induced by saturated fatty acids (SFAs). This finding is of particular interest because these conditions are thought to contribute to nonalcoholic fatty liver disease (NAFLD)^38^. Hepatic SIRT3 expression is significantly elevated in SFA-rich (palm oil) high-fat diet (HFD) conditions. SIRT3 overexpression strongly inhibits hepatic autophagy by downregulating AMPK via MnSOD, a known SIRT3 target. In addition, SIRT3 overexpression activates mammalian target of rapamycin complex 1 (mTORC1), which inhibits autophagy. The increased expression of SIRT3 promotes the susceptibility of palmitic acid/palm oil-induced hepatic injury. In contrast, SIRT3 deficiency induces autophagy and protects hepatocytes from lipotoxicity^38^.

Curiously, in other settings, SIRT3 can also act as a positive regulator of autophagy and mitophagy^37,39–41^. SIRT3 activates essential regulators of mitochondrial function, including peroxisome proliferator-activated receptor gamma coactivator 1-α (PGC-1α) and AMPK^36,37^. For instance, SIRT3 appears to protect neurons from ischemia, at least in part by inducing autophagy. SIRT3 decreases neuronal apoptosis in primary cultures of cortical neurons exposed to oxygen glucose deprivation (OGD) to mimic neuronal ischemia. SIRT3 increases AMPK activity and potentiates the induction of autophagy in OGD-treated neuronal cells. In addition, the overexpression of SIRT3 partially rescues mitochondrial function, including the generation of ATP and the mitochondrial membrane potential^39^. SIRT3-mediated mitophagy has also been demonstrated to protect tumor cells from apoptosis under hypoxic conditions^40^.

### The regulation of autophagy by SIRT6

SIRT6, a predominantly nuclear protein, deacetylates various proteins, including the histones H3K9 and H3K56^42^. Recent reports have demonstrated a protective role for SIRT6 against cardiovascular diseases^43–45^. SIRT6 reduces the formation of foam cells associated with early atherosclerosis (AS) in an autophagy-dependent manner^43^. The oxidized form of low density lipoprotein-cholesterol (ox-LDL) plays a role in the initiation and progression of AS^46^. Human monocytic THP1 cells treated with ox-LDL display increased lipid droplets in the cytoplasm. Treatment with ox-LDL reduces the level of SIRT6, which results in the inhibition of autophagy and decreases the levels of LC3 and Atg5. Foam cell formation induced by ox-LDL is ameliorated by the overexpression of wild-type SIRT6 but not by the expression of a catalytically inactive SIRT6 mutant (SIRT6 H133Y). In addition, SIRT6 overexpression preserves autophagic flux in THP1 cells treated with ox-LDL. This occurs in part because SIRT6 overexpression decreases the expression of miR-33, which increases autophagy and the expression of ABCA1 and ABCG1. This upregulation of ABCA1 and ABCG promotes cholesterol efflux and prevents the formation of macrophage foam cells^43^. As such, SIRT6-mediated autophagy plays a protective role in preventing atherosclerosis.

SIRT6 also suppresses isoproterenol (ISO)-induced cardiac hypertrophy through the activation of autophagy. ISO reduces autophagy and induces cardiac hypertrophy in primary neonatal rat cardiomyocytes (NRCMs) and Sprague-Dawley (SD) rats. SIRT6-mediated autophagy attenuates ISO-stimulated cardiomyocyte hypertrophy. SIRT6 also regulates cardiac autophagy in a FOXO3-dependent manner through the suppression of Akt signaling^44^. Moreover, autophagy induction by SIRT6 decreases the senescence of human bronchial epithelial cells (HBECs) induced by cigarette smoke extract (CSE). These effects appear to result from a SIRT6-dependent attenuation of the insulin-like growth factor-Akt-mTOR signaling axis^47^.

## The role of sirtuin-induced autophagy in diseases

### The role of sirtuin-mediated autophagy in cancer biology

Sirtuin-mediated autophagy is a potential therapeutic target for the treatment of cancers (Table 1). The regulation of autophagy by SIRT1 seems to play an important role in the development of gastric cancer. The high expression of SIRT1 and Beclin-1 (Atg6) is associated with shorter overall survival and reduced relapse-free survival in gastric cancer patients. In tumor gastric tumor samples, over 80% of malignant cells demonstrate increased autophagosome levels when compared to those of cells from adjacent normal tissue. Thus, the expression levels of SIRT1 and/or Beclin-1 are potential prognostic indicators for gastric cancer and are potential therapeutic targets for the disease^48,49^ (Table 1).

**Table 1:** The pathophysiological importance of mammalian sirtuins-mediated autophagy

Diseases	Sirtuins	Effects on autophagy	Effects on diseases	References
Cancer	SIRT1	Activation	Gastric cancer	^48, 49^
	SIRT2	Inhibition	Tumor suppression	^30, 31^
	SIRT3	Activation	Suppression	^40^
Kidney	SIRT1	Activation	Suppression of diabetic nephropathy	^51– 53^
	SIRT1	Activation	Decrease of renal cell apoptosis, inflammation and fibrosis	^54– 56^
Neuronal Diseases	SIRT1	Activation	Protection from neuronal damage or neurodegenerative diseases	^60– 73^
	SIRT2	Inhibition	Parkinson’s disease	^75, 77, 78^
	SIRT3	Activation	Protection form neuronal ischemia	^39^
Heart	SIRT1	Activation	Protection from atherosclerosis	^79^
	SIRT1	Activation	Protection from ischemia-reperfusion injury	^80^
	SIRT3	Activation	Reduction of myocardial hypertrophy	^81^
	SIRT3	Activation	Protection from diabetic cardiomyopathy	^82^
	SIRT6	Activation	Protection from cardiac hypertrophy	^44^
Liver	SIRT1	Activation	Prevention from ischemia-reperfusion injury after liver surgery	^83, 84^

Interestingly, sirtuin-dependent autophagy can also play a role in tumor suppression. As noted, SIRT2 deacetylates the transcription factor FOXO1, which regulates autophagy in human cancer cell lines in response to oxidative stress or serum starvation. In response to cellular stresses, the acetylation of FOXO1 is increased by dissociation from SIRT2. Furthermore, acetylated FOXO1 interacts with Atg7 to potentiate autophagic cell death. This FOXO1-dependent cell death appears to act as an important tumor suppressor mechanism in a human colon xenograft model^30^ (Table 1). In addition, SIRT2 suppresses basal autophagy, leading to mitotic catastrophe, an essential cell death mechanism that kills cancer cells in the setting of microtubule inhibitors^31^ (Table 1).

SIRT3 also mediates autophagy, including mitophagy, in response to hypoxia. Hypoxia increases the interaction between VDAC and Parkin. In cells subjected to hypoxia, SIRT3 deficiency results in reduced mitophagy, leading to further decreases in the mitochondrial membrane potential. This event subsequently increases the generation of reactive oxygen species (ROS), stimulating the degradation of the anti-apoptotic proteins Mcl-1 and survivin. Thus, the inhibition of *SIRT3* appears to promote apoptosis and enhances the sensitivity of cancer cells to hypoxia^40^ (Table 1).

Dysfunctional mitophagy and autophagy disrupt mitochondrial homeostasis. Mitochondrial dysfunction also causes metabolic reprogramming in response to environmental stresses. In addition, these bioenergetic changes associated with mitochondrial dysfunction alter stemness and cell fate decisions, which in turn impacts diseases such as cancer^50^.

### The role of sirtuin-mediated autophagy in kidney diseases

SIRT1-dependent autophagic induction in response to environmental stress is essential for the maintenance of renal function. Dysfunctional autophagy in the kidneys, including in cells such as podocytes, mesangial cells, endothelial cells, and tubular cells, contributes to the pathogenesis of diabetic nephropathy^51–53^ (Table 1). SIRT1, a positive regulator of autophagy, is also downregulated in diabetic kidneys^54^. In addition, numerous studies have suggested that SIRT1 protects against oxidative stress, reduces fibrosis and inflammation, and inhibits apoptosis in the kidneys^54–56^ (Table 1).

Protein acetylation plays a crucial role in the regulation of autophagy and can potentially be exploited as a potential new therapeutic target for the treatment of diabetic nephropathy^52,53,56–58^. In addition, hypoxia-mediated mitochondrial and renal damage in aged kidneys are prevented in a SIRT1-dependent autophagic process^59^. As noted, calorie restriction (CR) increases the level and activity of SIRT1^17^ and induces autophagy. Adult-onset and long-term CR in mice elevates SIRT1 expression in aged kidneys and attenuates hypoxia-associated mitochondrial and renal damage, at least in part by enhancing BCL2/adenovirus E1B 19-kDa-interacting protein 3 (Bnip3)-dependent autophagy. The deacetylation of FOXO3 by SIRT1 in CR increases the expression of Bnip-3 as well as that of p27, which results in increased autophagy and improved survival of primary renal proximal tubular cells (PTCs) under hypoxic conditions^59^.

### The role of sirtuin-mediated autophagy in neuronal diseases

Sirtuin-induced autophagy also has protective roles in the brain. Mammalian SIRT1 maintains brain integrity and protects against various neurodegenerative diseases, including Alzheimer’s and Parkinson’s disease^60–63^ (Table 1). SIRT1 promotes the growth and survival of neurons in the central nervous system by negatively regulating mTOR signaling, which in turn is a negative regulator of autophagy^64^. SIRT1 overexpression promotes neurite outgrowth in primary neurons and potentiates cell viability under nutrient stress conditions^64^. SIRT1-overexpressing neurons display increased tolerance to the accumulation of neurotoxic molecules such as β-amyloid (Aβ). SIRT1 overexpression decreases mTOR signaling by reducing the phosphorylation of mTOR at S2448 and the phosphorylation of p70S6K kinase at T289. In line with such findings, *Sirt1*-deficient mouse primary neurons display increased mTOR activity, which results in impaired cell survival and neurite outgrowth^64^. Consistent with these observations, the mTOR inhibitor rapamycin markedly improves neuronal cell survival in response to nutrient deprivation and significantly enhances neurite outgrowth in wild-type mouse neurons^64^ (Table 1).

SIRT1 also regulates the transcriptional activities as well as the acetylation status of essential neuroprotective proteins such as p53, nuclear factor kappa B, peroxisome proliferator-activated receptor γ (PPARγ), PPARγ coactivator-1α (PGC-1α), liver X receptor, and forkhead box O (FoxO)^61,62,65^. For instance, the overexpression of SIRT1 in the hippocampus of p25 transgenic mice, which exhibit massive degeneration of the forebrain with features of Alzheimer’s disease, significantly protects the mouse brain from neurodegeneration^61^. In addition, the treatment of these mice with resveratrol, an established SIRT1 activator, attenuates neurodegeneration in the hippocampus and prevents learning impairment by decreasing the acetylation of PGC-1α and p53^61,66^ (Table 1). The activation of SIRT1 in neurons by treatment with resveratrol deacetylates p53, and this reduces p53- and FOXO-dependent apoptosis. In addition, SIRT1 inhibits NF-κB signaling in microglia and astrocytes and protects AD neurons against Aβ-induced toxicity^62^. In Huntington’s disease, the mutant huntingtin protein inhibits the expression of PGC-1α, leading to the impairment of mitochondrial function. SIRT1 activates PGC-1α via deacetylation, which can act to reduce HD-associated mitochondrial impairment^62,67,68^ (Table 1).

The connection between sirtuins and autophagy might also be important for Parkinson’s disease. Resveratrol, a SIRT1 activator, induces autophagy and potentiates the degradation of α-synucleins in α-synuclein-expressing PC12 cell lines. The inhibition of AMPK decreases SIRT1 activity and reduces the beneficial effects of resveratrol on rotenone-induced apoptosis. Thus, AMPK and SIRT1 are required for resveratrol-mediated autophagy induction and for augmenting neuronal survival. In 1-methyl-4-phenyl-1, 2, 3, 6-tetrahydropyridine (MPTP)-treated mice (a model of Parkinson’s disease), resveratrol increases the activity of SIRT1 and thereby enhances the autophagic degradation of α-synuclein. In these MPTP-treated mice, resveratrol increases the survival of dopaminergic neurons, augments dopamine levels, and ameliorates behavioral impairments^69^. In simple model organisms such as yeast, the regulation of Atg32 by Sir2 prevents α-synuclein-induced toxicity during aging^70–72^. In addition, the regulation of autophagy by SIRT1 plays a protective role against prion-induced neuronal cell death and related neurodegenerative disorders^73^ (Table 1).

In sporadic Parkinson’s disease, unusual microtubule-dependent transport, mitochondrial dysfunction, and impaired autophagy have been reported. Changes in NAD^+^ metabolism in cells derived from sporadic Parkinson’s disease patients activate SIRT2, which in turn deacetylates α-tubulin, a SIRT2 target. The pharmacological or genetic inhibition of SIRT2 increases the level of acetylated α-tubulins. *SIRT2* deficiency facilitates trafficking by preventing microtubule network disruption and clearing misfolded proteins or protein aggregates^74–76^. Therefore, the negative regulation of autophagy by SIRT2 has the potential to be a therapeutic target for neurodegenerative diseases, including sporadic Parkinson’s disease^75,77,78^ (Table 1).

SIRT3 induces autophagy during neuronal ischemia. SIRT3 regulates mitochondrial energy metabolism, including ATP generation, and has protective roles in neuronal survival following oxidative stress. SIRT3 is upregulated during oxygen and glucose deprivation (OGD), a neuronal ischemia mimetic. The ectopic expression of SIRT3 markedly reduces OGD-induced lactate dehydrogenase release and neuronal apoptosis. These cells also display a decrease in hydrogen peroxide (H_2_O_2_) production, increases in ATP generation, and the maintenance of mitochondrial membrane potential (MMP). The overexpression of SIRT3 in cortical neurons dramatically increases the phosphorylation of AMPK and the subsequent induction of autophagy. Moreover, phosphorylated mTOR levels are decreased, providing another stimulus for autophagic induction. This suggests that SIRT3-induced autophagy is regulated by an AMPK-mTOR signaling axis, which represents a potential therapeutic target for neuronal protection^39^ (Table 1).

### The role of sirtuin-mediated autophagy in heart diseases

The regulation of autophagy by the sirtuin family appears to play a beneficial role in protection against cardiovascular diseases (Table 1). For instance, *SIRT1* deficiency increases oxidative stress, inflammation, and foam cell formation and augments the senescence of endothelial cells, vascular smooth muscle cells, and monocytes/macrophages. Autophagy deficiency also contributes to vascular aging and atherosclerosis^79^ (Table 1). The overexpression of SIRT1 protects mouse heart tissues against ischemia-reperfusion (I/R) injury. In these mice, autophagy is stimulated, FOXO3 signaling is activated, nitric oxide synthase is hyperphosphorylated, and NF-kB is deacetylated^80^ (Table 1).

SIRT3-dependent autophagy also appears to play a role in cardiac hypertrophy. The induction of autophagy as well as the deacetylation of FOXO1 by SIRT3 reduces myocardial hypertrophy following treatment with angiotensin II. For instance, the overexpression of SIRT3 in the cardiomyocyte H9C2 cell line increases autophagy and attenuates cardiomyocyte hypertrophy induced by angiotensin II^81^. However, *Sirt3* knockout mice display impaired autophagy and increased angiotensin II-induced myocardial hypertrophy^81^ (Table 1).

SIRT3 also has a pivotal cardioprotective role in the setting of diabetic cardiomyopathy in part by activating Parkin-mediated mitophagy^82^ (Table 1). Streptozotocin (STZ) exposure causes diabetes mellitus-induced myocardial damage and interstitial fibrosis in mice. In addition, STZ-treated mice display increased apoptotic cardiomyocyte death and mitochondrial injury with increases in the number of abnormally shaped mitochondria. The effect of STZ is more pronounced in *Sirt3*-deficient mice than in wild-type mice. In *Sirt3* knockout mice, deacetylated FOXO3A levels and Parkin expression levels are decreased. SIRT3 overexpression in neonatal mouse cardiomyocytes increases autophagy and protects against mitochondrial injury and cardiomyocyte apoptosis. As such, a SIRT3-FOXO3A-Parkin-mediated mitophagy pathway appears to play key protective roles in moderating diabetic cardiomyopathy^82^ (Table 1).

SIRT6 is another important mammalian sirtuin protein that appears to protect against cardiac hypertrophy. Cardiac hypertrophy induced by isoproterenol significantly decreases autophagy, which is regulated in part by SIRT6^44^. *SIRT6* overexpression increases autophagy in primary neonatal rat cardiomyocytes and in rat hearts. Thus, the cardioprotective effect of SIRT6 in an isoproterenol (ISO)-stimulated cardiac hypertrophy model appears to be dependent on the induction of autophagy. SIRT6 dramatically increases the nuclear accumulation of the FOXO3 transcription factor possibly via attenuating Akt signaling. The positive regulation of autophagy by SIRT6 in isolated cardiomyocytes and intact rat hearts protects against ISO-induced cardiac hypertrophy^44^ (Table 1).

### The role of sirtuin-mediated autophagy in liver diseases

Autophagy regulation by SIRT1 contributes to protecting against liver damage by maintaining basic hepatic function^83,84^(Table 1). Mortality after liver surgery is largely dependent on ischemia/reperfusion (I/R) injury. After I/R injury in the liver, SIRT1 expression is highly reduced. The ectopic expression or increased activity of SIRT1 dramatically restores defective autophagy, reduces the onset of the mitochondrial permeability transition, and mitigates hepatocyte death after I/R injury. Mechanistically, SIRT1 interacts with mitofusin-2, a target of the SIRT1 deacetylase. Furthermore, SIRT1 overexpression moderately induces autophagy in wild-type cells but not in *MFN2*-deficient cells. Thus, *Sirt1* deficiency results in a series of sequential events, including defective autophagy, mitochondrial dysfunction, and hepatocyte death after I/R^84^ injury (Table 1).

## The physiological roles of sirtuin-induced autophagy

### The effect of sirtuin-induced autophagy on lifespan

Sirtuins and autophagy generally have lifespan-extending effects^6,85,86^. Dietary-restricted model organisms, such as yeast, worms, flies, and mice, live longer due to an increase in SIRT1 activity as well as the induction of autophagy. In addition, the overexpression of SIRT1 or specific autophagy genes in model organisms, including mice, and the administration of autophagy-activating or sirtuin-regulatory small molecules such as rapamycin, resveratrol, or spermidine increase lifespan^87–89^.

Mitophagy contributes to a wide range of physiological processes, including aging and lifespan. Patients with ataxia telangiectasia have defects in ATM kinase, a master regulator of DNA damage and a facilitator of DNA double-strand break repair. In mice and worms, ATM-deficient neurons display significantly increased PARylation, low NAD^+^ levels, and mitochondrial dysfunction. Augmenting intracellular NAD^+^ levels reduces the severity of A–T neuropathology, normalizes neuromuscular function, delays memory loss, and extends lifespan in both worms and mice. Increased intracellular NAD^+^ levels also lead to neuronal DNA repair and improve mitochondrial quality via mitophagy^90^, although the precise role of NAD-dependent sirtuins in this process is incompletely understood.

### The effect of sirtuin-induced autophagy on inflammation and senescence

SIRT1-dependent autophagy blocks endothelial inflammation. Resveratrol, a SIRT1 activator, alleviates tumor necrosis factor (TNF)-induced inflammation by increasing autophagy in human umbilical vein endothelial cells (HUVECs)^91^. The genetic inhibition of autophagy using siRNAs targeting *ATG5* or *BECN1* suppresses the downregulation of inflammatory factors, including ICAM1, PTGS2, and MMP9, through treatment with resveratrol. Again, autophagy is required for the suppression of TNF-induced inflammation in a SIRT1-dependent manner and protects vascular endothelial cells against inflammation. Resveratrol also increases cyclic AMP content and the expression of PKA and SIRT1 as well as the activity of AMPK. Overall, resveratrol reduces endothelial inflammation by inducing autophagy, which is regulated by the activation of a cAMP-PKA-AMPK-SIRT1 signaling pathway^91^.

The activation of autophagy by sirtuins also attenuates cellular senescence. The expression levels of SIRT6 are decreased in lung homogenates from chronic obstructive pulmonary disease patients^47^. Cigarette smoke extract (CSE) reduces SIRT6 expression in human bronchial epithelial cells (HBECs). The CSE-induced senescence of human bronchial epithelial cells is inhibited by SIRT6 overexpression, and SIRT6 knockdown and deacetylase-defective mutant SIRT6 (H133Y) enhance the senescence of HBECs. SIRT6 overexpression induces autophagy via the attenuation of IGF-Akt-mTOR signaling. SIRT6-induced autophagy prevents senescence by CSE^47^. SIRT6 is involved in CSE-induced HBEC senescence via autophagy regulation, which contributes to the attenuation of IGF-Akt-mTOR signaling. In addition, the negative regulation of SIRT1-dependent autophagy, which is targeted by miR-212, augments cellular senescence^92^.

### The effect of sirtuin-induced autophagy on stem cell functions

Recent reports have indicated that SIRT1 and autophagy regulate stem cell fate decisions and protect stem cells from diverse stresses, such as hypoxia, serum deprivation, and oxidative stress^93–95^. Oxidative stress further increases the level of apoptosis in *Sirt1−/*− murine embryonic stem cells (ESCs) compared to that in wild-type cells. In both *Sirt1*-deficient murine ESCs and *SIRT1* knockdown human ESCs, autophagy is markedly decreased in response to oxidative stress, as measured by the decreased conversion of LC3-I to LC-II, a low level of Beclin-1 protein, and reduced LC3 puncta. In addition, the class III PI3K/Beclin-1 and mTOR pathways are partially required for the protective roles of SIRT1-dependent autophagy in ESCs under oxidative stress conditions^95^.

*Sirt1* deficiency also causes impaired mitochondrial dynamics in ESCs. The generation of intracellular ROS by high concentrations of exogenous H_2_O_2_, in combination with the autophagy inhibitor 3-methyladenine (3-MA), further increases apoptosis in *Sirt1−/*− murine ESCs compared with wild-type ESCs. Cellular oxidative stress leads to the disruption of the mitochondrial membrane potential. Treatment with H_2_O_2_, in conjunction with 3-MA treatment, results in mitochondrial depolarization in wild-type cells. However, the combinatorial treatment of 3-MA with H_2_O_2_ in *Sirt1*−/− cells does not cause an increased loss of membrane potential because *Sirt1*−/− leads to impaired autophagy. Thus, autophagy appears to regulate the mitochondrial membrane potential in response to H_2_O_2_ in a Sirt1-dependent manner^95^.

## Concluding remarks and future directions

**Fig. 2:**
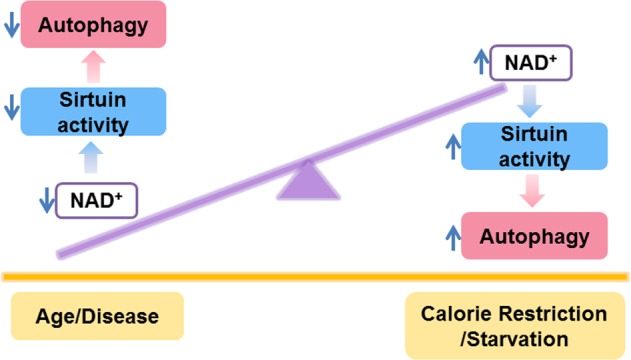
A potential model for the connection between NAD^+^, sirtuins and autophagy. Nutrient limitation increases the level of the essential cellular metabolite NAD^+^, which activates sirtuins by inducing autophagy within cells and tissues. In contrast, the activity of sirtuins and autophagy decreases during normal aging, as well as due to some diseases^26^, and correlates with the known reduction in levels of tissue NAD^+^

Although the mechanisms remain incompletely understood, as outlined above, the functional links between autophagy and sirtuins play essential roles in diverse cellular processes, including aging and various age-related diseases. Both sirtuins and autophagy function as sensors and effectors of nutrient depletion and appear to integrate this information into a coordinated response to maintain cellular homeostasis. The question therefore arises as to why this relationship between the sirtuin family and autophagy evolved? While no definitive answer for this question exists, it is tempting to speculate that autophagy arose, in part, to allow organisms to resist metabolic stresses, including starvation. After all, starvation is a classic stimulus of autophagic induction. Nonetheless, the enzymatic core machinery of autophagy (i.e., Atg proteins such as Atg5, Atg7, etc.) has little intrinsic capacity to monitor metabolic conditions. In contrast, sirtuins, the enzymatic activity of which is dependent on the NAD/NADH ratio and the enzymatic reaction of which involves acetyl-CoA, are perfectly positioned to act as energy rheostats. Under nutrient limiting conditions, NAD^+^ levels rise^26,27^, activating sirtuin proteins, which in turn can deacetylate the autophagy machinery. The observation that the autophagy machinery is highly acetylated under basal conditions might reflect high acetyl-CoA conditions, which occur when nutrients are available. Under this scenario, starvation induces sirtuin activity in an NAD^+^-dependent manner, triggering the deacetylation of the core autophagy machinery (Fig. 2). This deacetylation results in two independent events that help the organism withstand nutrient stress, namely, unlocking the enzymatic activity of the autophagy machinery and liberating acetyl-CoA. Although speculative, this scenario is consistent with the growing appreciation that cytosolic acetyl-CoA levels play an important role in autophagy^96^.

The link between sirtuins and autophagy may explain one other important aspect of regulation that was intimated in this review. It is generally believed that autophagic flux slows down as organisms age^86,97,98^. Recent evidence has suggested that this is also true for more specialized forms of autophagy, such as mitophagy^99^. The molecular basis for this phenomenon is not understood. Again, it is tempting to hypothesize that the well-known age-dependent decline in NAD^+^ levels might provide a basis for this effect. Under this scenario, a primary fall in tissue levels of NAD^+^ would limit sirtuin activity and thereby limit autophagic flux (Fig. 2). Given the growing interest in NAD biology^6,26,27,100^, it will be important in the future to determine whether NAD supplementation exerts its beneficial effects through sirtuin-dependent autophagy induction. In addition, since direct sirtuin-activating compounds (STACs), including resveratrol and SRT2104, and indirect activators, such as nicotinamide riboside, are currently in clinical trials for the treatment of cardiovascular diseases, cancer, diabetes, and neuropathy^26,101^, it is critical to more fully understand how these agents work. Again, it is tempting to hypothesize that their clinical benefits might result from the link between sirtuin activity and autophagic flux. Finally, the small molecule activation of sirtuins and hence the indirect activation of autophagy might be advantageous over the direct and constitutive activation of autophagy. This supposition is based on the growing literature that autophagy might provide a survival advantage for tumor cells^102,103^. In such a scenario, there may be safety concerns for the long-term administration of direct autophagy inducers. In contrast, therapeutic strategies that exploit the interaction between sirtuins and the autophagy machinery might provide for a more regulated and physiological approach. Undoubtedly, a fuller understanding of the cross-regulation between sirtuins and autophagy will likely provide important insights and potentially novel therapeutic avenues to combat a host of age-related diseases, as well as potentially a strategy to slow aging itself.
